# Quantifying the Ebbinghaus figure effect: target size, context size, and target-context distance determine the presence and direction of the illusion

**DOI:** 10.3389/fpsyg.2015.01679

**Published:** 2015-11-04

**Authors:** Hester Knol, Raoul Huys, Jean-Christophe Sarrazin, Viktor K. Jirsa

**Affiliations:** ^1^Institut de Neurosciences des Systèmes, Aix-Marseille UniversitéMarseille, France; ^2^Institut de la Santé et de la Recherche Médical, UMR_S 1106Marseille, France; ^3^Systems Control and Flight Dynamics Department, Office National d'Etudes et de Recherches Aérospatiales (ONERA)Salon de Provence, France; ^4^Centre National de la Recherche ScientifiqueParis, France; ^5^Centre de Recherche Cerveau & Cognition - UMR5549, Université Toulouse III - Paul SabatierToulouse, France

**Keywords:** perception, visual illusions, ventral stream, dorsal stream, Ebbinghaus illusion

## Abstract

Over the last 20 years, visual illusions, like the Ebbinghaus figure, have become widespread to investigate functional segregation of the visual system. This segregation reveals itself, so it is claimed, in the insensitivity of movement to optical illusions. This claim, however, faces contradictory results (and interpretations) in the literature. These contradictions may be due to methodological weaknesses in, and differences across studies, some of which may hide a lack of perceptual illusion effects. Indeed, despite the long history of research with the Ebbinghaus figure, standardized configurations to predict the illusion effect are missing. Here, we present a complete geometrical description of the Ebbinghaus figure with three target sizes compatible with Fitts' task. Each trial consisted of a stimulus and an isolated probe. The probe was controlled by the participant's response through a staircase procedure. The participant was asked whether the probe or target appeared bigger. The factors target size, context size, target-context distance, and a control condition resulted in a 3 × 3 × 3+3 factorial design. The results indicate that the illusion magnitude, the perceptual distinctiveness, and the response time depend on the context size, distance, and especially, target size. In 33% of the factor combinations there was no illusion effect. The illusion magnitude ranged from zero to (exceptionally) 10% of the target size. The small (or absent) illusion effects on perception and its possible influence on motor tasks might have been overlooked or misinterpreted in previous studies. Our results provide a basis for the application of the Ebbinghaus figure in psychophysical and motor control studies.

## Introduction

**Figure 1:**
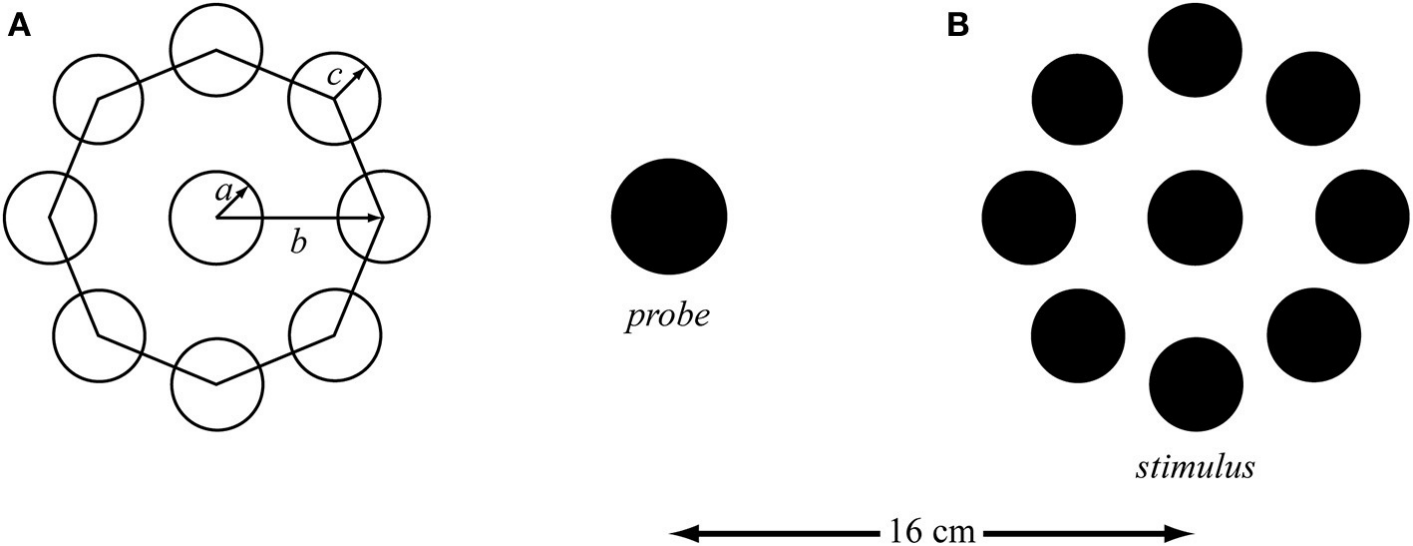
**(A)** The parameters of the Ebbinghaus figure with the radius of the target (*a*) and the context (*c*), and the distance from the target center to the context center (*b*). **(B)** Example of the Ebbinghaus stimulus with the scaling probe (not scaled to real size). The distance between the center of the probe and the center of the target was 16 cm. The context circles covered approximately 75% of the circumference.

Optical illusions evoke a perceived image, color, contrast, lightness, brightness, or size that differs from the physical “reality” of the figure. These illusions have mainly been used to test theories predicting the successes and failures of the perceptual system, particularly by the Gestalt school (Robinson, 1998). Optical illusions have been classified based on the behavioral manifestation of 45 illusions (e.g., Coren et al., 1976). One commonly mentioned class is the one of size-contrast illusions, in which the size of an element is affected by its surrounding elements. A famous size-contrast illusion is the Ebbinghaus figure (see Figure 1), also called Titchener circles.

For over a century the Ebbinghaus figure has been used in experimental psychology to evoke an optical illusion of the perceived circle size. The Ebbinghaus figure consists of a target circle (*a* in Figure 1A) that is surrounded by multiple context circles (*b* in Figure 1A). It is thought that by surrounding the target with small or big circles, the target will appear bigger or smaller, respectively (Obonai, 1954; Massaro and Anderson, 1971). More than 10 theories have been trying to explain the physiological mechanism(s) responsible for the over- and under-estimation of the target (for a review see Robinson, 1998). However, attempts to quantify the illusion magnitude of this widely used geometrical visual illusion have not resulted in a (complete set of) geometrical rule(s), which is in all likelihood at least partly due to the broad spectrum of parameters involved. Several rules have been developed to identify the principal factors influencing the perceptual judgment evoked by the Ebbinghaus figure (e.g., Massaro and Anderson, 1971; Roberts et al., 2005; Nemati, 2009). Principle factors that have been identified are the size of the target (*a* in Figure 1A), the context circle size (*c* in Figure 1A), the number of context circles (Massaro and Anderson, 1971; Roberts et al., 2005), the target-context distance (*b* in Figure 1A; Roberts et al., 2005; Im and Chong, 2009) and the size of the area of empty space between the context circles (Nemati, 2009). However, these proposed rules do not specify the exact interplay between the three parameters specified in Figure 1A, which makes utilization of these rules for parameter selection and the prediction of the corresponding illusion effect tricky if not impossible. Furthermore, these rules have barely been validated. Indeed, Franz and Gegenfurtner (2008) concluded their review stating that: “…currently not much is known on the exact sources of the Ebbinghaus illusion.”

This lacuna did not withhold experimentalists to employ this figure to shed light on the so-claimed distinction between the ventral and dorsal visual pathway (see the review of Franz and Gegenfurtner, 2008). Accordingly, the visual system contains two distinct streams: the ventral pathway is specialized in processing information leading to conscious perception whereas the dorsal pathway is specialized in processing information for sensory-motor action (Goodale and Milner, 1992; Milner and Goodale, 1995). The dorsal stream encodes visual information into the required coordinates for skilled motor behavior, and does this in absolute metrics determined relative to the observer (egocentric frame of reference), whereas the ventral stream encodes the information into object properties relative to the properties of other objects (scene based frame of reference), and therefore provides a rich and detailed representation (Goodale, 2014). Based on this hypothesis, online control, and the programming of movements would recruit the dorsal stream and, since absolute metrics are determined relative to the observer and not relative to the context of the object, would therefore be insensitive to visual illusions (Milner and Goodale, 2008; Goodale, 2014).

Several studies have reported evidence for the illusion insensitivity during grasping movements (Aglioti et al., 1995; Haffenden et al., 2001; Milner and Goodale, 2008; Stöttinger et al., 2010, 2012). However, these findings seem to mismatch with studies that show a clear effects of visual illusions on grasping (Pavani et al., 1999; Franz et al., 2000) and pointing (Gentilucci et al., 1996; van Donkelaar, 1999). These seemingly contradicting results led to the hypotheses (for a review see Franz and Gegenfurtner, 2008) that a clear functional dissociation between perception and action cannot be made (Gentilucci et al., 1996; Franz et al., 2000), that the ventral pathway would have to be partially involved (Aglioti et al., 1995; Carey, 2001), and that two dorsal pathways (e.g., the use and grasp system) exist in stead of one (Binkofski and Buxbaum, 2013).

Seemingly conflicting results of studies that quantified the illusion effect in perception and movements tasks may be explained in various methodological ways (Bruno et al., 2008; Bruno and Franz, 2009). Franz (2001) classified two measurement types, to which he referred as the standard and the non-standard perceptual measures. In the standard method, participants either compare the size of two illusion stimuli or of one probe and one illusion stimulus. In the non-standard method, participants scale the aperture (with or without vision of the hand) to indicate the perceived size. Potential problems arising in the standard method are: First, by changing the size of the inner circle of an Ebbinghaus figure, as in Aglioti et al. (1995), it is not just the target size that is changed but also the distance from the target to the context circle, and therefore also the illusion magnitude (Roberts et al., 2005). Second, sometimes a stimulus-stimulus configuration is used in the perceptual task whereas a stimulus-probe configuration is used in the motor task (as in Aglioti et al., 1995). Third, if a task consists of comparing stimulus A with stimulus B, the question comes up which stimulus evokes an illusion effect (if any). For the non-standard method, a potential problem is that it is questionable that studying the perceptual illusion effect by asking participants to scale their aperture indeed provides a “pure” perceptual measure. Note that this method has generated conflicting results (Daprati and Gentilucci, 1997; Haffenden and Goodale, 1998). Across methods, if graspable targets are used (in the perceptual task), the minimum stepsize of the target or probe might be relatively big compared to the illusion magnitude. Furthermore, Franz and Gegenfurtner (2008) identified methodological biases and statistical corrections in the comparison of perception and movement task data. There are, however, also studies that have not quantified or reported the illusion effect on perception (e.g., van Donkelaar, 1999; Jackson and Shaw, 2000; Westwood et al., 2000; Ellenbürger et al., 2012), or have not used a control condition (Ellenbürger et al., 2012). To recapitulate, the conflicts in the reported results may well be due to the various methods used, and potential weakness therein as discussed here above. Consequently, it is hard, if possible at all, to draw strong conclusions about the proposed dissociation of the ventral and dorsal stream in perceptuomotor tasks based on research using optical illusions.

With the aim to (partly) fill this gap, we here provide a fully parameterized Ebbinghaus figure, and systematically quantified the illusion effect for parameter ranges that are relevant for behavioral experiments. Thereto, we used a methodology that is well-established in the psychophysics literature, namely, the staircase procedure. We predicted that target size, context size, and target-context distance would affect the perceived target size of the Ebbinghaus figure, but that some parameter combinations, in particular those involving small target sizes (Massaro and Anderson, 1971), would fail to elicit a significant illusion effect. Intuitively, we further expected that some stimulus configurations, in particular those evoking a strong illusion effect, would be perceptually more distinct than others, and that this would affect the decision making as expressed in the response times. That is, we expected response time to scale inversely with perceptual distinctiveness. Our results will be able to guide future experimentalists, which, we hope, will contribute in clarifying the role of the ventral stream in the guidance of motor behavior.

## Materials and methods

### Participants

Twelve participants (6 females and 6 males, age mean ± SD = 28.9 ± 3.5) with normal or corrected to normal vision volunteered in the experiment. The experiment was performed in accordance with the Helsinki Declaration and all participants gave a written informed consent prior to their participation.

### Apparatus

The visual stimuli were drawn and generated using the Psychophysics Toolbox in Matlab R2009b (The MathWorks Inc., Natick, MA) (Brainard, 1997; Kleiner et al., 2007). Black stimuli were presented against a white background (see Figure 1) and multisampled to control for aliasing effects. To prevent interference from previous trials and to control hemispace bias the stimuli were randomly presented on the left or the right side of the screen while an isolated probe (i.e., target without context circles) was presented simultaneously on the opposite side of the screen at a distance of 16 cm from the stimulus (and at the same height). The stimuli were displayed with a Dell Precision T3610 and Nvidia Quadro K2000 video card on a Dell P2714H monitor with a resolution of 1920 × 1080 pixels (597.9 × 336.3 mm, 52.96 × 29.27°) and a frame rate of 60 Hz. The participants sat at a 60 cm distance from the monitor and their heads were supported with a chin-rest so as to ensure that the distance between the head and the monitor remained fixed.

### Procedure

Based on a fully geometrical description (Figure 1A), three target sizes (2 × *a* in Figure 1A), three target—context distances (*b* in Figure 1A), three context sizes (bigger, equal, and smaller than the target; *c* in Figure 1A), and three control conditions (isolated targets) were selected, resulting in a 3 × 3 × 3 + 3 factorial design. The equidistantly spaced context circles covered approximately 75% of the circumference in all conditions to control for the completeness of the surround (Roberts et al., 2005). Consequently, the number of context circles varied as a function of context size and target-context distance. The stimuli diameters were 0.5, 1.0, and 2.0 cm [These sizes were chosen with an eye on planned future studies involving Fitts' task; the corresponding indices of difficulty (i.e., ID = log_2_(2*D*/*W*), where *D* and *W* represent the distance between the targets and the target width, respectively; Fitts, 1954 were 6, 5, and 4, respectively.) Context sizes were 20, 100, and 180% of the target size; i.e., 0.1, 0.5, and 0.9 cm for the small target, 0.2, 1.0, and 1.8 cm for the medium target, and 0.4, 2.0, and 3.6 cm for the big target. Three distances from the center of the target to the center of the context circles (i.e., *b* in Figure 1A) were calculated based on the smallest distance being 10% bigger than the radius of the target plus the radius of the biggest context; i.e., 0.8 cm for the small target, 1.6 cm for the medium target, and 3.0 cm for the big target. The other two distances were incremented with 0.6 cm for each distance. All dimensions were corrected for pixel size and rounded to the nearest integer.

A two-down, one-up staircase procedure was used to find the perceptual threshold between the probe and the target in which the probe size was adjusted (García-Pérez, 1998). Two staircases per condition were used, one in which the initial condition of the probe was 0.4 cm bigger than the target size, and one in which it was 0.4 cm smaller. Each staircase started with a probe diameter step size of four pixels (i.e., 0.12 cm). The participants were tasked with pressing a key (*A* or *L*) for the bigger appearing target or probe on the left (*A*) or right side (*L*) of the keyboard corresponding to the target and probe location on the screen. Depending on the response of the participant, the probe size was adjusted according to the two-down, one-up staircase procedure. In a sequence of responses, a reversal is the event where the response to probe n deviates from that at n-1. After each reversal the step size was halved, until the minimum of one pixel (i.e., 0.03 cm) was reached, which was then retained. The participants were instructed to respond as soon as they had decided which key to press, but it was made clear that it was not a reaction time task. After each key press, the stimulus disappeared and a random noise window was displayed for 1 s followed by a fixation cross (duration: 0.5 s). Then the next stimulus with the adjusted probe appeared. A staircase was terminated and removed from the cue after a participant had reversed the direction of the staircase 11 times. After five conditions, the participants could take a small pause. Upon completing the first half of the experiment, the participants took a 10–15 min break. The entire experiment lasted for about 2 h.

### Data analysis

From the last 10 reversals, the perceptual threshold (*PT*) was calculated according to Equation (1),

(1) $$\begin{array}{l} PT=\frac{1}{m}\sum_{j=1}^{m}\left(\frac{1}{n}\sum_{i=1}^{n}SC_{up_{i}}+\frac{1}{n}\sum_{i=1}^{n}SC_{low_{i}}\right) \end{array}$$

in which *m* corresponds to the number of staircases (here Equation 2), *n* represents the number of reversals taken into account (here 10), Explicitly, the mean of *SC*_*up*_ and *SC*_*low*_ are calculated based on the last 10 reversals and are referred to as the upper and lower staircase threshold, respectively. The range between the mean *SC*_*up*_ and mean *SC*_*low*_ reflected the area of uncertainty (*AU*) (Equation 2).

(2) $$\begin{array}{l} AU=\frac{1}{n}\sum_{i=1}^{n}SC_{up_{i}}-\frac{1}{n}\sum_{i=1}^{n}SC_{low_{i}} \end{array}$$

To control for inter-individual differences in the judgment of the target sizes in the control condition, and to allow for inter-individual and inter-trial comparisons, the judgments were corrected by subtracting the perceptual threshold of the control trial (*PT*_*control*_) from the corresponding perceptual threshold of each trial (*PT*_*trial*_), i.e., *IM* = *PT*_*control*_ – *PT*_*trial*_, where *IM* stands for illusion magnitude. For the statistical analyses and the visualizations, the illusion magnitude was used.

Response time was defined as the time between stimulus presentation onset and the participant's response. We next computed the average response time before a participant crossed one of the staircase thresholds for the first time (referred to as *RT*_*base*_). For this procedure, the first response was omitted. The average response time following this threshold crossing was referred to as *RT*_*AU*_.

Three-Way repeated measures ANOVAs with target size (*a* in Figure 1A), distance (*b* in Figure 1A), and context size (*c* in Figure 1A) as within participants factors were performed to investigate the effects on the illusion magnitude and the area of uncertainty. If significance levels were met (α = 0.05), the tests were followed up by Bonferroni *post-hoc* tests (α = 0.05). A Four-Way repeated measures ANOVA with target size (*a*), distance (*b*), context size (*c*), and response moment (*RT*_*base*_, *RT*_*AU*_) as within participants factors was used to investigate significant effects on response time. The degrees of freedom were corrected according to the Greenhouse-Geisser method to control for non-sphericity of the data if necessary. If this was the case, the adjusted degrees of freedom were reported below. In order to examine if the perceived size of the targets of the illusion trials were significantly different from those of the control trials, a paired samples *t*-test was performed for each condition. Pearson correlation coefficients were calculated to investigate potential (linear) correlations between response time, area of uncertainty and illusion magnitude, and between the response time before the area of uncertainty for the upper and lower staircase.

## Results

### Illusion magnitude

**Figure 2:**
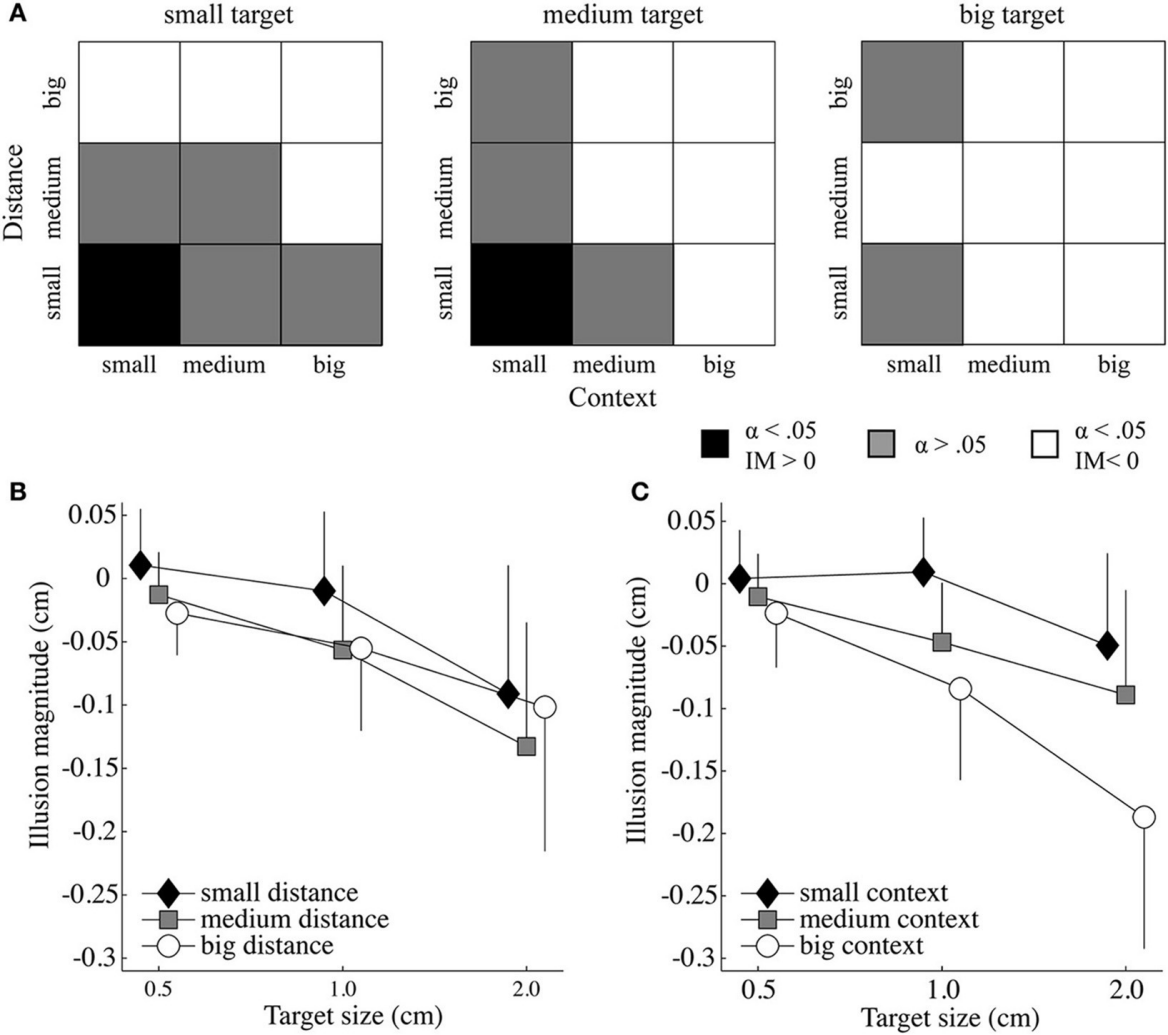
**Illusion effects as a function of target size, context size, and target-context distance.(A)** Significance levels resulting from paired samples *t*-tests and the direction of the illusion magnitude (IM) for each target size as a function of the context-target distance and context size. The black and white squares indicate a significant effect for bigger perceived targets and smaller perceived targets, respectively. The gray squares show conditions that were not significantly different from the control trials (α = 0.05). **(B)** Mean IM (and standard deviation) as a function of target size and distance. **(C)** Mean IM (and standard deviation) as a function of target size and context size.

Recall, for the statistical analysis the control perceptual threshold (*PT*_*control*_) per target size was subtracted from the *PT*_*trial*_ to control for the participants' ability to judge targets of different sizes. Figure 2A displays the results of the paired samples *t*-tests to investigate if the illusion magnitudes were significantly different from the control trials. There, it can be seen that a target appeared only bigger than it was when the context and distance were small (i.e., 20% of the target size and 110% of target plus biggest context size, respectively) and the target size small or medium (i.e., 05 or 1.0 cm). In 33% of the cases, there was no significant illusion effect. For all other conditions the target was perceived as smaller than it actually was.

Significant main effects for illusion magnitude were found for context size [*F*_(2, 22)_ = 40.698, *p* = 0.000, $\eta_{p}^{2}=0.787$], distance [*F*_(2, 22)_ = 24.181, *p* = 0.000, $\eta_{p}^{2}=0.687$] and target size [*F*_(1.244, 13.686)_ = 28.973, *p* = 0.000, $\eta_{p}^{2}=0.725$]. The illusion magnitudes of all target sizes were significantly different [all *p* < 0.005; mean ± SD for the small (−0.01 ± 0.01), medium (−0.04 ± 0.01), and big (−0.11 ± 0.02) target size], as well as for all context sizes [all *p* < 0.001; mean ± SD for the small (−0.01 ± 0.01), medium (−0.05 ± 0.01), and big (−0.10 ± 0.02) context]. For target—context distance, small distances differed significantly from the medium (*p* < 0.000) and big distances (*p* < 0.005), however, medium and big distances did not differ significantly from each other [*p* > 0.05; mean ± SD for distance small (−0.03 ± 0.01), medium (−0.07 ± 0.01), and big (−0.06 ± 0.01)].

The analysis further revealed a significant interaction between target size and target-context distance [*F*_(4, 44)_ = 3.933, *p* = 0.008, $\eta_{p}^{2}=0.263$; see Figure 2B], as well as between target size and context size [*F*_(2.244, 24.687)_ = 12.822, *p* = 0.000, η_*p*_^2^ = 0.538; see Figure 2C], indicating that context size and distance influenced the illusion effect differently for the different target sizes. When significantly different from the baseline, the big and medium target-context distances always had a diminutive effect on the perceived target size (Figure 2A). The illusion magnitude under the small distance was always smaller than that of the medium and big distance, except when the illusion had a magnifying effect on the perceived target size. Except for the small distance, the big context size always had a stronger diminutive effect on the perceived target size than the medium context size, and the medium context size always had stronger diminutive effect than the small context size (see Figure 2C). The interaction of the three factors distance, context and target size approached significance [*F*_(3.857, 42.431)_ = 2.427, *p* = 0.065, $\eta_{p}^{2}=0.181$]. The target-context distance by context size interaction was not significant.

### Area of uncertainty

The area of uncertainty was only significantly influenced by target size [*F*_(1.260, 13.855)_ = 22.731, *p* = 0.000, η_*p*_^2^ = 0.674]. *Post-hoc* tests indicated that it increased in the control conditions as well as in the illusion trials as target size increased [for illusion trials: big vs. medium or small target size (*p* < 0.005), medium vs. small target size (*p* < 0.05)].

### Response time

**Figure 3:**
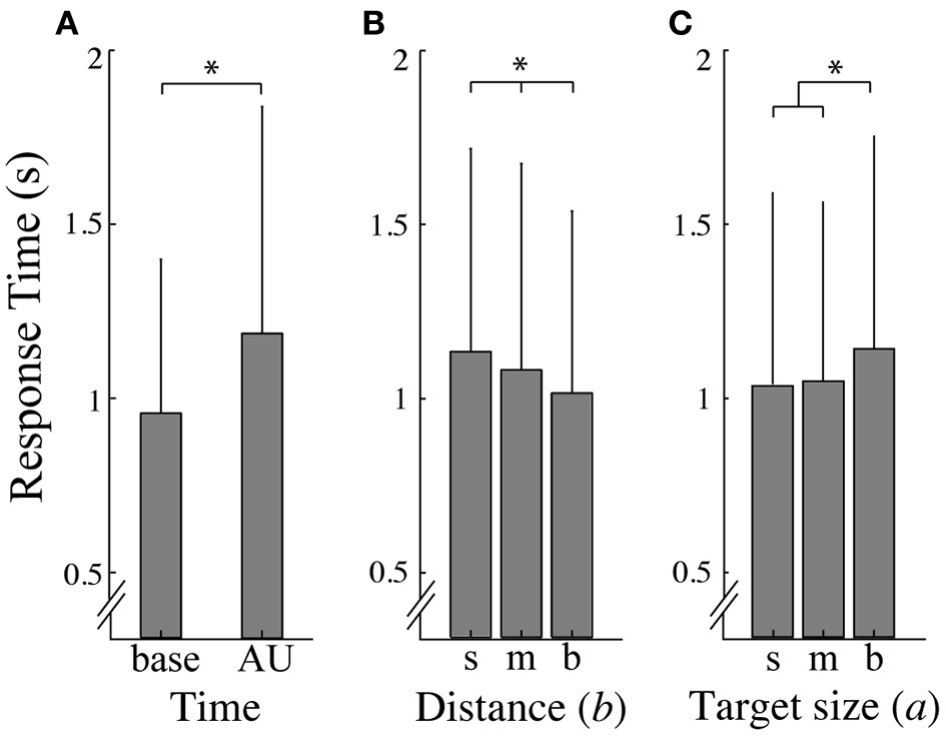
**Response times**. **(A)** Average response time (and standard deviation) as a function of time; *base* refers to the baseline responses and *AU* refers to responses in the area of uncertainty. **(B, C)** represent the average response time (and standard deviation) over a small (*s*), medium (*m*), and big (*b*) distance **(B)** and target size **(C)**. Asterisks indicate significant effects (α = 0.05).

The response times for the three target sizes for the baseline (*RT*_*base*_*)* and area of uncertainty (*RT*_*AU*_*)* control conditions were not significantly different [*p* > 0.05; mean ± SD *RT*_*AU*_ for target small (0.89 ± 0.38), medium (0.89 ± 0.47), and big (0.96 ± 0.57)]. Presentation of the Ebbinghaus figures, however, provoked longer response times compared to the control conditions [*F*_(1, 11)_ = 35.795, *p* = 0.000, η_*p*_^2^ = 0.765]. In addition, for the illusion trials, *RT*_*AU*_ was significantly higher than *RT*_*base*_ [*F*_(1, 11)_ = 7.8, *p* = 0.017, η_*p*_^2^ = 0.415; Figure 3A]. Further, a significant main effect of target-context distance [*F*_(2, 22)_ = 6.1, *p* = 0.008, η_*p*_^2^ = 0.356; Figure 3B] and of target size [*F*_(2, 22)_ = 4.9, *p* = 0.17, η_*p*_^2^ = 0.310; Figure 3C] on the response time was found. *Post-hoc* tests revealed that response times were significantly longer at small distances compared to big distances (*p* < 0.01) and in the big target size conditions than in the small target size conditions (*p* < 0.05). Furthermore, an interaction effect between target size and distance was found [*F*_(4, 44)_ = 2.9, *p* = 0.034, η_*p*_^2^ = 0.207] which was mainly caused by the medium distance. For the small and big distance, the response times increased with increasing target size, whereas for the medium distance the response time was shortest at the medium target size.

### Correlations between illusion magnitude, area of uncertainty, and response time

**Figure 4:**
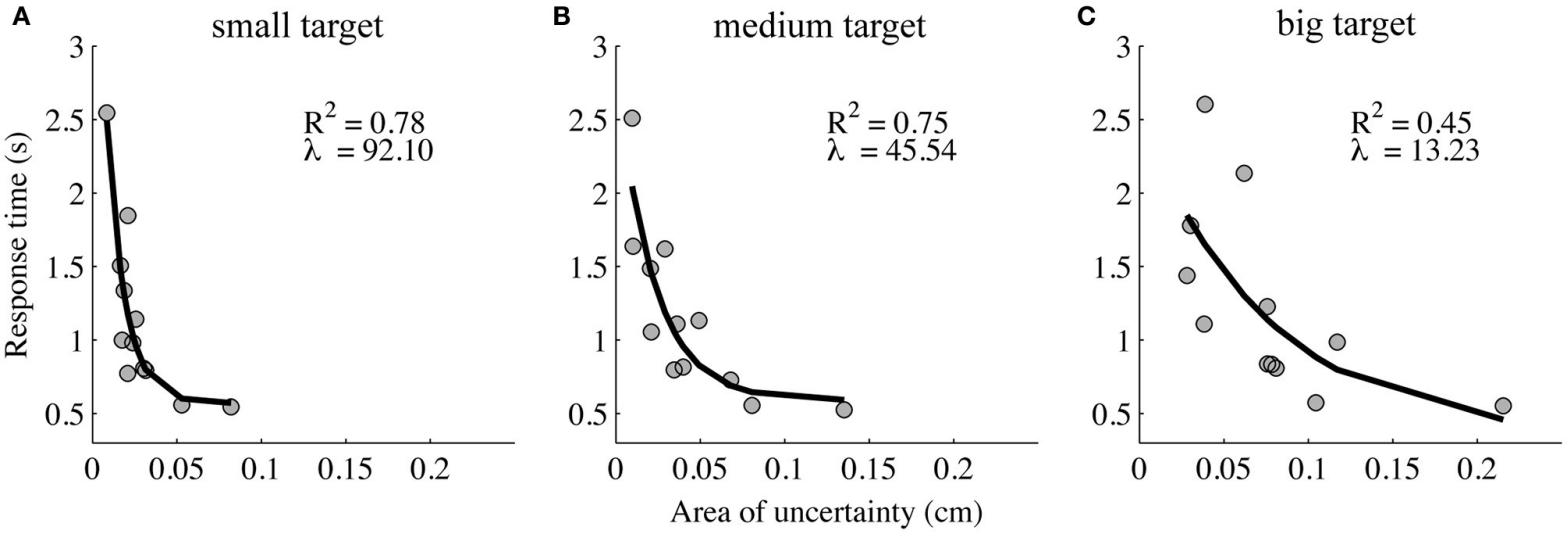
**Response time in the area of uncertainty as a function of the area of uncertainty**. The response time decays exponentially as a function of the area of uncertainty, and the decay increases with the increasing target size (the small **(A)**, medium **(B)**, and big target **(C)** are represented in the left, middle, and right panel, respectively).

A significant but weak correlation was found between the absolute illusion magnitude and the area of uncertainty [*r*_(322)_ = 0.12, *p* < 0.05]. Further, as the absolute illusion magnitude increased, the response time (moderately) increased [*r*_(322)_ = 0.25, *p* < 0.001]. In contrast, if the area of uncertainty increased, the response time decreased [*r*_(322)_ = −0.41, *p* < 0.001]. Further examination of the relation between the area of uncertainty and response time across participants revealed that it was exponential, and that the exponent decreased with target size (Figure 4).

## Discussion

### Summarize findings

We investigated the role of context size, target-context distance, and (actual) target size on perceived target size using a staircase procedure. In accordance with our hypotheses, we found no significant illusion effect in 33% of the 27 applied parameter combinations. Whenever there was an illusion effect, all three factors affected the PT. A target circle appeared bigger in only two out of 27 conditions (i.e., 7%), namely, when presenting a small or medium target with small context circles at a small distance. In all other cases (i.e., 60%) the target appeared smaller. The area of uncertainty grew with a growing target size and with a decreasing target-context distance. Furthermore, the response time increased whenever context circles surrounded the target, and with increasing target size. The response time correlated positively with the illusion magnitude, but opposing our prediction, correlated negatively (but weakly) with the area of uncertainty.

#### Illusion magnitude

Massaro and Anderson (1971) formulated an equation according to which the illusion effect scales positively with target size. In accordance therewith, the authors reported two experiments that both showed increased illusion effects as a function of increasing target size (more specifically, 1.3, 1.5, and 1.7 cm). Our findings are in agreement with theirs, and we showed that this effect holds for a wider range of target sizes (namely, 0.5, 1.0, and 2.0 cm).

Nemati (2009) argued that illusionary effects of the Ebbinghaus figure are the result of a combination of a size contrast effect and the area of empty space (i.e., the area of the stimulus that is not filled by the context). The size contrast effect holds that smaller or bigger context circles, relative to the target, cause an over- or under-estimation, respectively, of the perceived target size due to contrast mechanisms (Massaro and Anderson, 1971). If so, our findings should reflect only size contrast effects since we controlled for the empty space area by covering 75% of the circumference in all stimulus configurations. In accordance with Roberts et al. (2005), we reported, however, that small context circles did not always make the target appear bigger (i.e., only in 22% of the cases a target with a small context was perceived as being bigger). That is, the Ebbinghaus figure cannot be reduced to “just” a size-contrast effect in which a target is always perceived as being bigger when the context is smaller than the target size. In other words, we oppose earlier work describing magnifying and reducing effects of the smaller and bigger surround on a target, respectively (Obonai, 1954; Massaro and Anderson, 1971).

As compared to Roberts et al. (2005), fewer parameter combinations resulted in positive illusion magnitudes (i.e., over-estimation of target size) and, furthermore, the absolute maximum illusion magnitude was bigger. Differences in the direction and size of the illusion effect could possibly be explained by the different target sizes (Roberts et al., 2005 employed target sizes of 1.05 and 1.4 cm whereas we used 0.5, 1.0, and 2.0 cm), since target size played a big role in the size of the illusion magnitude, and interacted with target-context distance and context size.

Target-context distance has been suggested to be more important than the size-contrast effect for the illusion magnitude (Im and Chong, 2009). This suggestion, however, is not supported by our results: although a significant effect of target-context distance on illusion magnitude was found, this effect was weaker than the effect of context size and target size.

Whereas a target-context distance larger than 1.9 cm (3.5°) was found to decrease the perceived target size (Roberts et al., 2005), a small target-context distance (0.3–1.2 cm in Girgus et al., 1972) has been shown to increase the perceived target size (Oyama, 1960; Girgus et al., 1972). That is, perceived target size seems to reveal an inverted u-shape as a function of context distance. In line therewith we found increased perceived target sizes for small distances (0.8 and 1.6 cm for the small and medium sized target, respectively) when combined with a small context whereas a distance of 1.4 cm in combination with a small context size did not result in an increased perceived target size. For all other target-context distances (i.e., 2.0–4.2 cm), if there was an effect, the perceived target size was smaller than the actual target size. However, this was also the case for the smallest target-context distance for the biggest target (2.4 cm). In fact, the largest target was never perceived as being bigger, which could be due to the target-context distances that for this target size always exceeded the 0.3–1.6 cm range, or other protocol variations (*a, b* in Figure 1). An increase in distance up to 3.6 cm (all conditions except the large distance—large target condition) resulted in a larger illusion magnitude. A distance greater than 3.6 cm (i.e., 4.2 cm; large distance—large target) reduced the illusion magnitude (see Figure 2B), which could explain the interaction effect between target size and target-context distance. That is, these findings agree with an inverted u-shape of the illusion magnitude over target-context distance. This might stroke with what Sarris (2010) called Ebbinghaus' law of relative size-contrast, in which he describes a general inverted u-shape trend for size-contrast effects (Note, though, that Ebbinghaus pointed at the relative size comparison of dwarfs, men, and dolls; Ebbinghaus and Dürr, 1902; Sarris, 2010). However, to confirm this hypothesis, a broader range with smaller and larger target-context distances should be tested.

We did not find a significant interaction effect between target-context distance and context size. In Roberts et al. (2005), this interaction was tested for in two experiments. In their experiment 3, which was similar to our experiment—they reported an illusion magnitude from 0.084 to 0.12 cm for a target size of 1.4 cm, a context size of 0.35 and 1.4 cm, and a target-context distance of 1.05–4.67 cm—the interaction turned out to be significant whereas in the other (experiment 1) it did not. Our results indicate that the illusion magnitude was affected by target-context distance and context size in a similar fashion (illusion magnitude of 0.1–0.13 cm for target size 1 and 2 cm, context size 0.2–3.6 cm, and a target-context distance of 1.6–4.2 cm).

#### Area of uncertainty

We quantified the distance between the points as asymptotically reached by the upward and downward staircases, and refer to it as the area of uncertainty. The area of uncertainty represents a measure of the perceptual distinctiveness of the illusion. We showed that it increased as the target size increased. This might be a simple demonstration of Weber's law (or the Weber-Fechner law) according to which sensitivity to changes in perception decreases when stimulus intensity increases (i.e., the ratio between the “just-noticeable difference” in a physical property and its magnitude is invariant). Schmidt et al. (1979) proposed that variability (in force production) would increase proportionally with the absolute magnitude (of the forces) (Schmidt et al., 1979). Along the same line, it might be that the variability represented by the area of uncertainty scales linearly with target size. Interestingly, it has been shown that internal noise increases with letter size (Pelli and Farell, 1999). It may well be that our scaling of target size similarly increased internal noise. In that regard, investigating variations in internal noise as a function of the various Ebbinghaus figure parameters (Figure 1A), as well as relative to control conditions, may well shed novel insights into the (strength of the) illusion effect and its perceptual distinctiveness.

The increase of the area of uncertainty confirms the use of a minimum of two staircases and shows the directionality imposed by the procedure. By taking the mean of the two staircases, information about the distance between these two staircases is lost, even though this contains valuable information about the perceptual and decision-making processes, and thus the illusion effect.

#### Response time

Our response time data showed a complex effect of the illusion. First of all, the response time in the control conditions was unaffected by target size, which stands in contrast to reports of an inverse relation between target size and reaction time (Payne, 1967; Osaka, 1976; Marzi et al., 2006). The illusion conditions, however, showed two contrasting effects: the influence of target size on the response time (response time increased with target size; Figure 3C), and the influence of target-context distance on the response time (response time decreased with increasing target-context distance; Figure 3B). Furthermore, the response time correlated positively (but weakly) with the absolute illusion magnitude and negatively with the area of uncertainty. Since, to our knowledge, most of the Ebbinghaus studies neglected the response time, we can only refer to a study with schizotypal traits in which the authors measured the illusion magnitude and the response time of two Ebbinghaus figures (small and big context circles with a fixed target size and target-context distance; Bressan and Kramer, 2013), and reports of simple reaction time studies (Sperandio et al., 2010). Whereas Bressan and Kramer found that individuals with a longer response time tended to show less illusion effects (Bressan and Kramer, 2013), others reported that the reaction time was shorter when the target appeared bigger/longer (Sperandio et al., 2010; Ponzo illusion: Plewan et al., 2012). We found that strong illusion effects went hand in hand with long response times. Thus, rather than being scaled according to the perceived target size, we found that the response time scaled with the (absolute) illusion magnitude. It may be that, at least to some extent, these discrepancies are due to methodological differences: in the reaction time studies quickness of response was stressed and the illusions were presented briefly only (ranging from 10 to 250 ms), unlike our study. Regardless, the question remains what the origin of the increase in response time is, and how response time and illusion magnitude causally relate (if so). Given the widely accepted view that response time somehow reflects the cognitive processes involved in a given performance, and the more easily comprehendible effects relative to the control condition and the moment of assessing it (i.e., baseline vs. the area of uncertainty), we believe that response time, which is typically discarded in studies using visual illusion as a means to investigate the ventral-dorsal visual pathway distinction, may provide an interesting novel entry point to its effects. We will return to this issue in the section below.

### Models describing the ebbinghaus illusion

Until now it has not been possible to predict the illusion magnitude given a certain set of parameters. Massaro and Anderson (1971) and Roberts et al. (2005) described a simple model of the Ebbinghaus illusion. The model developed by Massaro and Anderson (1971), to which they refer as judgmental model, is based on the idea that the Ebbinghaus figure works as a simple size-contrast illusion with a fixed number of context circles. They did not take into account that the completeness of the surroundings would influence the illusion magnitude as previously shown (Massaro and Anderson, 1971; Roberts et al., 2005). Nemati (2009) extended the hypothesis of Massaro and Anderson (1971) with the idea that the area of empty space influences the magnitude and direction of the illusion effect. By controlling for the completeness of the surroundings, as in Roberts et al. (2005), we controlled for differences in the empty space. The sole remaining explaining factor, thus, would accordingly be the size-contrast effect. As said above, this was not the case. Roberts et al. (2005) proposed a model according to which the illusion magnitude scales exponentially with inducer distance. Their model could not explain our data in 78% of all factor combinations. We incorporated three times the number of participants, and should therefore have shown an exponential decaying trend if the model would have been correct.

**Figure 5:**
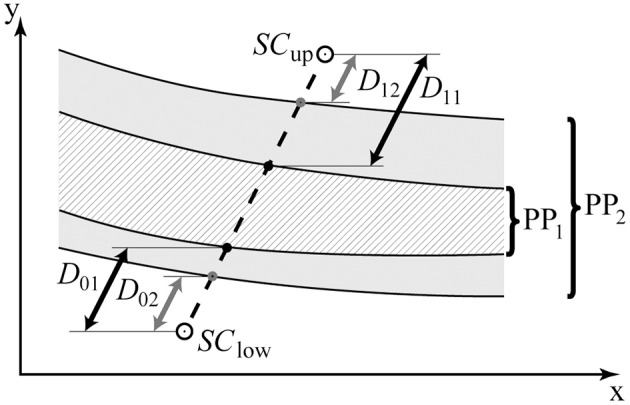
**Cartoon illustration of the Ebbinghaus figure parameter space**. PP_1_ and PP_2_ contain the area of uncertainty for two different participants; the black (*D*_01_, *D*_11_) and gray arrows (*D*_02_, *D*_12_) represent the corresponding distances to the area of uncertainty from the start of the two staircases (*SC*_up_, *SC*_low_), respectively. The non-shaded vs. shaded areas (PP_1_, PP_2_) may represent distinct regimes in parameter space in which perceptual decision-making is deterministic vs. random, respectively.

In that regard, a potential shortcoming of existing models is that they do not allow for non-linear effects like hysteresis, multi-stability, etc. Dynamical systems are described in the space spanned by its state variables. If one stable solution exists in that space (an attractor), the system will invariantly evolve toward it. If multiple stable solutions exist (multi-stability), it will evolve toward one of the attractors, dependent on the initial conditions. In a bifurcation, the number and/or nature of the system's solution changes when the so-called bifurcation parameter is (gradually) scaled. Hysteresis only occurs in multi-stable systems, and refers to the phenomenon that when changing a bifurcation parameter the system's history determines to which stable attractor the system will evolve. Such effects are the hallmark of non-linear systems, and evidence that behavioral, perceptual, and cognitive systems belong to that class of non-linear systems abounds (e.g., Haken et al., 1985; Tuller et al., 1994; van Gelder, 1998; see also Kelso, 1995 for a review). Our present results only hint at the existence of non-linear effects (note that the experiment was not designed so as to reveal them). The response time data and, in particular their exponential decay as a function of the size of the area of uncertainty (Figure 4), may provide indications that are consistent with non-linear effects. Within the borders of the area of uncertainty, the responses are at chance level. Outside this area of uncertainty the participants perceive a clear difference between the target and the probe. This observation is open to interpretation in terms of the existence of two distinct “states” or regimes (multi-stability). In this sense, the borders of the area of uncertainty are linked to the bistability regime of the coexistent two distinct states (see Figure 5). They are, however, not synonymous therewith. Intuitively, it makes sense to assume that response time scales with the degree of (perceptual) uncertainty. Consequently, the shorter an observer's distance to the area of uncertainty, the slower her/his response. In the present staircase procedure, the participants' initial conditions were the same, but the size of their area of uncertainty varied. In other words, their distance to the area of uncertainty, which scales inversely with its size, was different (see Figure 5). Consistent with our present argument, the results in Figure 4 indicate an exponential relation between the size of the area of uncertainty and response time. For the argument to hold, however, a similar trend should exist for the participants individually. We tested this in two ways: first, for each participant we calculated the distance to the area of uncertainty for the upper and lower staircase and the corresponding response times for the second response. For most of the participants (11 out of 12), the distance was larger for the upper staircase and the response times were shorter. Both effects were significant (paired *t*-test; both *p* < 0.001). Second, we linearly regressed each participant's response times against the distance to the area of uncertainty. Unfortunately, due to the high variability, only three out of 12 regressions were significant at α = 0.05. Their average slope was –1.23. Regardless, all regressions had a negative slope; the mean slope of the non-significant regressions was –0.49. That is, across participants the response time tended to decrease as the area of uncertainty increased. In combination, these results argue in favor of a relation between the distance to the area of uncertainty and response time, and are suggestive of the existence of distinct regimes of operation. Clearly, however, future efforts will be needed to either falsify or reject this idea.

### Illusion effects in motor tasks

The combination of an increased illusion magnitude, standard deviation (as suggested by visual inspection of Figures 2B,C), and the increase in response time as target size and, concomitantly, the area of uncertainty decreased, might indicate that strong illusion effect evoking parameters induce instability in the participants' decision-making. But which processes underlie this change in stability is uncertain. As discussed in Section Models Describing the Ebbinghaus Illusion, possibly the area of uncertainty and the longer response time hint at hysteresis. If hysteresis indeed exists, then the mechanism underlying the change of strength of the illusion effect is linked to multistability and transitions from one state to another. The parameter space in Figure 2 offers a starting point to develop experimental paradigms, in which the Ebbinghaus illusion is used to drive parametrically coordination behavior through a “perceived” parameter such as size in contrast to the “physical” parameter, the actual size. It should be noted, however, that it should not be naively assumed that the parameter space of the illusion effects in Figure 2 is the same, when the Ebbinghaus illusion is used in sensorimotor coordination experiments. This assumption holds, from the dynamical system perspective, only for weak coupling of the perception-action system. Weak coupling means that two dynamic systems, when coupled, display the same qualitative dynamics as in absence of coupling, and undergo changes that can be regarded as small perturbations. For instance, two systems that display oscillations in absence of coupling can realize arbitrary relative phase relations; when weakly coupled, they still display oscillations, but now only certain relative phase relations are stable, others unstable. For strong coupling, the intrinsic oscillation may disintegrate and different behaviors may occur that cannot be understood anymore through the notion of relative phase. This limitation should be kept in mind when developing applications of the Ebbinghaus illusion parameter space, in which the perception-action coupling, if strong, may alter the system dynamics significantly.

How visual illusion figures affect perception and action has previously been shown to be a complex puzzle, and highly depending on the research method and selected parameters (Bruno and Franz, 2009). In the present perceptual study, the effect sizes and direction of the effects resulting from the perception of the Ebbinghaus figure appeared to be highly dependent on the selected parameters. Observed illusion effects, if present, up to (exceptionally) 10% of the target size might explain why illusion effects in motor tasks have sometimes failed to materialize.

Fitts' law predicts the time required to rapidly move between two targets as a ratio of the width of the target and the distance to the target (Fitts, 1954). The Ebbinghaus (like) figure has been implemented in a Fitts' task to test whether a perceptual illusion would affect the motor behavior (van Donkelaar, 1999; Fischer, 2001; Ellenbürger et al., 2012). van Donkelaar (1999) and Ellenbürger et al. (2012) found that movement was affected by the illusion (in terms of movement time (van Donkelaar, 1999); van Donkelaar, dwell time, and harmonicity Ellenbürger et al., 2012). However, Van Donkelaar and Ellenbürger and colleagues did not quantify the illusion magnitude of their Ebbinghaus figures. In contrast, Fischer (2001) found an effect of context size and context-target distance on perception but no effect on movement (at least, in the absence of stimulus-movement delays). The perceptual effects, while significant, were rather small; they ranged from –0.3 to 0.2 mm, that is, about an order of magnitude smaller than the range reported here. It remains to be seen, however, to which degree the method used by Fischer to quantify the illusion effect on perception, namely scaling a probe until it matches the perceived target size, provides robust results (see also Introduction). In fact, we found no illusion effect in 33% of the parameter combinations for a similar target size as in Fischer's study (1 vs. 1.2 cm, respectively). Thus, if the reported perceptual results fail to be robust, the results of Fischer's movement study might simply be due to the lack of illusion effects. Furthermore, since the illusion magnitude was often found to be relatively small, it might be that the measures used for motor studies were too coarse to capture small effects of the illusion. In conclusion, studies like these hamper drawing firm conclusions on how perceptual and motor effects relate, and to what degree the ventral and dorsal stream operate in a functionally distinct manner.

### Methodological concerns

Both the Ebbinghaus figure and the staircase procedure can be adapted by changing numerous parameters such as parameters *a, b, c* in the Ebbinghaus figure (Figure 1A), and the (adaptive) stepsize, procedure, starting point, and number of reversals for the staircase procedure. Due to the contradictory results of various methods to quantify the illusion effect, and due to the large number of Ebbinghaus figure configurations tested in this study, the widely studied and applied two-up, one-down staircase procedure was chosen, which is a two alternative forced choice method (2AFC). Several previous studies also applied the staircase procedure to study different features of the Ebbinghaus figure (Roberts et al., 2005; Im and Chong, 2009; McCarthy et al., 2013). Another version of the 2AFC method to study perception is the method of constant stimuli, in which a fixed number of combinations of (Ebbinghaus) figures are shown a certain number of times in a random order. In this case, the sampling is random and every possible stimulus-pair combination is presented equally often. This method allows for the full sampling of a so-called psychometric function. The slope and the horizontal shift of this psychometric function (i.e., a cumulative probability distribution) and the X_50_ value (also called the Point of Subjective Equality) then specify the illusion effect. A big area of uncertainty might be linked to a shallow slope of the psychometric function, and the PT should be equal to the point of subjective equality. McCarthy et al. (2013) have performed 4 experiments with using both the staircase procedure (experiment 2) and the method of constant stimuli (experiments 1, 3, and 4) showing that both methods result in similar points of subjective equality. Considering the long history of staircase procedures in the field of psychophysics (García-Pérez, 1998), and the magnitude of the illusion effect being in a similar range as in the similar study of Roberts et al. (2005) the staircase procedure opens new doors in order to quantify the Ebbinghaus illusion effect in a systematic way.

Clearly, this method has its own limitations and assumptions. For example, to which percent-larger responses (referred to as percent-correct responses in visual contrast and luminance studies) the staircases converge using different protocols, is still under debate (for a review see García-Pérez, 1998). At chance level, a two-up, one-down procedure will bias responses in the “up” direction rather than the “down” direction. However, a two-up, one-down procedure also assures a better precision than a one-up, one-down procedure (García-Pérez, 1998). Two staircases that start from positions bigger and smaller than the actual target size, assure a fully symmetrical procedure, and a bias in both the “up” and “down” direction, for the upper and lower staircase, respectively. Anyhow, regardless these limitations, visual illusion research, be it in the context of the visual stream dissociation or otherwise, may benefit from these (and potentially other) more or less standardized and in-depth investigated methods.

## Conclusion

Concluding, since the Ebbinghaus figure is widely used but no clear rule is set, inter-comparison of the broad range of parameters remains difficult. We haven shown that the illusion magnitude highly depends on an interplay of target size, context size and target-context distance, and that a third of the parameter combinations here used did not evoke an illusion effect. Importantly, however, even if the group-averaged illusion magnitude can be predicted by a set of stimulus configuration parameters (or established rules), the predictive value for individual performances would likely be limited given the marked inter-individual variability. Thus, the implementation of the Ebbinghaus figure in various fields of research needs to be handled with care and quantified per study.

### Conflict of interest statement

The authors declare that the research was conducted in the absence of any commercial or financial relationships that could be construed as a potential conflict of interest.
